# Evaluating the indicators of the elder-friendly city in Urmia 

**Published:** 2019-08

**Authors:** Marjan Hosseinpour

**Affiliations:** ^*a*^Maternal and Childhood Obesity Research Center, Urmia University of Medical Sciences, Urmia, Iran.

## Abstract

**Background:**

According to the World Health Organization, the number of elderly people over 60 will double ‎by 2025. Despite the increasing percentage of elderly people in communities, most urban ‎spaces, environments, and houses are designed without taking into account the needs of the ‎elderly. Due to factors such as a relative decline in birth rates and increased life expectancy, it ‎seems that in the near future, the elderly population in Iran will form a significant part of the ‎demographic structure of the country. The main solution to meet the needs of the elderly is to ‎obtain the standards of an elder-friendly city. Therefore, this research was conducted with the ‎aim of evaluating indicators (With the emphasis on social, cultural, recreational and health ‎indicators) of the Elder- friendly City, in Urmia. ‎

**Methods:**

This descriptive cross-sectional study was carried out in 50 elderly over 60 years old in different ‎regions of Urmia, Feb. 2019. Data collection was done by the WHO questionnaire. In terms of ‎reliability, Cronbach s alpha coefficient was 78%, which is acceptable. Data were analyzed by ‎Stata software. Systematic sampling was performed from 2 nursing homes in Urmia. Eligible ‎criteria were the residence in Urmia and age above 60 years. The Kolmogorov-Smirnov test was ‎used to check the normality of the data and t-test to compare the mean of the indicators.‎

**Results:**

A total of 50 elderly people participated in this study. Their average age was 67.1 ± 7.32 and ‎‎51% were women. The results showed that all social, cultural and educational indicators in ‎Urmia are fairly appropriate and in concordance with the standard, but health indicators are ‎somewhat lower. ‎

**Conclusions:**

Health indicators are an important part of elder-friendly city standards. Therefore, further ‎studies and appropriate interventions are recommended to obtain the standard level of health ‎indicators.‎

**Keywords:**

Age-friendly communities, Health, Urmia, Iran

